# Electronic Data Capture System (REDCap) for Health Care Research and Training in a Resource-Constrained Environment: Technology Adoption Case Study

**DOI:** 10.2196/33402

**Published:** 2022-08-30

**Authors:** Irma Adele Maré, Beverley Kramer, Scott Hazelhurst, Mapule Dorcus Nhlapho, Roy Zent, Paul A Harris, Michael Klipin

**Affiliations:** 1 Department of Surgery, School of Clinical Medicine Faculty of Health Sciences University of the Witwatersrand Johannesburg South Africa; 2 Division of Biomedical Informatics and Translational Science Wits Health Consortium Johannesburg South Africa; 3 School of Anatomical Sciences Faculty of Health Sciences University of the Witwatersrand Johannesburg South Africa; 4 Sydney Brenner Institute for Molecular Bioscience Faculty of Health Sciences University of the Witwatersrand Johannesburg South Africa; 5 School of Electrical & Information Engineering University of the Witwatersrand Johannesburg South Africa; 6 Division of Epidemiology and Biostatistics, School of Public Health Faculty of Health Sciences University of the Witwatersrand Johannesburg South Africa; 7 Division of Nephrology and Hypertension Department of Medicine Vanderbilt University Medical Center Nashville, TN United States; 8 Department of Biomedical Informatics Vanderbilt University Medical Center Nashville, TN United States; 9 Department of Biomedical Engineering Vanderbilt University Nashville, TN United States; 10 Department of Biostatistics Vanderbilt University Medical Center Nashville, TN United States

**Keywords:** electronic data capture, implementation science, Research Electronic Data Capture, REDCap, biomedical informatics, South Africa

## Abstract

**Background:**

Electronic data capture (EDC) in academic health care organizations provides an opportunity for the management, aggregation, and secondary use of research and clinical data. It is especially important in resource-constrained environments such as the South African public health care sector, where paper records are still the main form of clinical record keeping.

**Objective:**

The aim of this study was to describe the strategies followed by the University of the Witwatersrand Faculty of Health Sciences (Wits FHS) during the period from 2013 to 2021 to overcome resistance to, and encourage the adoption of, the REDCap (Research Electronic Data Capture; Vanderbilt University) system by academic and clinical staff. REDCap has found wide use in varying domains, including clinical studies and research projects as well as administrative, financial, and human resource applications. Given REDCap’s global footprint in >5000 institutions worldwide and potential for future growth, the strategies followed by the Wits FHS to support users and encourage adoption may be of importance to others using the system, particularly in resource-constrained settings.

**Methods:**

The strategies to support users and encourage adoption included top-down organizational support; secure and reliable application, hosting infrastructure, and systems administration; an enabling and accessible REDCap support team; regular hands-on training workshops covering REDCap project setup and data collection instrument design techniques; annual local symposia to promote networking and awareness of all the latest software features and best practices for using them; participation in REDCap Consortium activities; and regular and ongoing mentorship from members of the Vanderbilt University Medical Center.

**Results:**

During the period from 2013 to 2021, the use of the REDCap EDC system by individuals at the Wits FHS increased, respectively, from 129 active user accounts to 3447 active user accounts. The number of REDCap projects increased from 149 in 2013 to 12,865 in 2021. REDCap at Wits also supported various publications and research outputs, including journal articles and postgraduate monographs. As of 2020, a total of 233 journal articles and 87 postgraduate monographs acknowledged the use of the Wits REDCap system.

**Conclusions:**

By providing reliable infrastructure and accessible support resources, we were able to successfully implement and grow the REDCap EDC system at the Wits FHS and its associated academic medical centers. We believe that the increase in the use of REDCap was driven by offering a dependable, secure service with a strong end-user training and support model. This model may be applied by other academic and health care organizations in resource-constrained environments planning to implement EDC technology.

## Introduction

### Background

#### Challenges to Electronic Data Capture Implementation in Health Care

Electronic data capture (EDC) and management is a vital part of the administrative process in almost all industries, but despite its many advantages, adoption in the health care research and clinical service delivery domains has lagged behind other industries [1-13]. Major obstacles include cost, lack of policy at management level, implementation failure, and data security concerns [1-7]. In addition, research information applications developed specifically for one environment usually require significant resources and support to function in another environment because the technology, support, security, and privacy needs are often different [5,8-10]. Many institutions in sub-Saharan Africa and other low- and middle-income countries lack the technical resources to support information systems for health care research and clinical service delivery [11-13]. Moreover, power and network infrastructure may be unreliable [11-13].

Even with the necessary organizational and infrastructure support, the success of EDC software applications is not guaranteed [4,5,14]. The lack of domain knowledge in resource-constrained environments such as sub-Saharan Africa has hampered the implementation of EDC technologies [11]. There is often a paucity of individuals with the necessary clinical, academic, and IT skills required to support critical health care data management systems [11]. Field-workers and clinician scientists, although highly skilled and valued in their respective domains, may not be well versed in technology for the capture, storage, and transmission of health data [4-7].

Once infrastructure and skills-resourcing issues have been overcome, familiarity with deeply ingrained systems and processes at every level of the research enterprise is a natural cause of resistance to change [1,4-7,15-18]. This includes, for example, the replacement of hard-copy files with electronic data collection instruments for clinical research informatics. Strategies to obviate the resistance to implementation of new technology include demonstrating trustworthiness and benefits of the technology, while at the same time easing the transition through access to appropriate training and support [1,5-7,16,17].

#### The State of EDC at the University of the Witwatersrand Faculty of Health Sciences Before Research Electronic Data Capture Adoption

The University of the Witwatersrand (Wits) is a research-intensive university based in the metropolitan area of Johannesburg, South Africa, an upper middle–income environment [19]. The Wits Faculty of Health Sciences (FHS) operates within a health care system weakened by sociopolitical and historical issues and strained by an ongoing quadruple burden of disease [12,20,21]. Setting up systems to support academics in their clinical and research activities is subject to budget limitations and constrained by the geographical distribution of the approximately 2500 health sciences staff and 7000 students, spread over 3 discrete academic teaching platforms and many field sites in both urban and rural regions [22,23].

Before the implementation of the centrally supported EDC system at the University of the Witwatersrand Faculty of Health Sciences (Wits FHS) and the associated research entities in 2012, electronic data management services were fragmentated, inconsistent, and variable. Individuals and research entities were using local devices and legacy systems familiar to them or choosing new products to implement based only on their own needs, abilities, and budget. This fragmentation was not desirable from an organizational perspective because the data sets were isolated, the financial and human resources used for procurement and management were diluted, and the security and privacy of data could not be guaranteed [5,9,10].

During the same period, most of the patient health record data being collected at Wits FHS–related medical centers were paper-based. Clinical staff were burdened by service delivery demands [20] and restricted in the time they had available for research or data capture [24]. Where electronic research data collection instruments were used, they ranged from simple spreadsheet programs such as Microsoft Excel to enterprise-wide data management systems. At the time, there were no preferred instruments within the Wits FHS, and the safety, security, and privacy of the data were at the discretion of individual researchers. There was little standardization of metadata or clinical coding systems, and, as a result, interoperability and secondary analysis of data were rare. This affected patient care and limited the dissemination of important knowledge gained in treating various infectious diseases and diseases of lifestyle that continue to burden the South African health care system [12,20,21].

#### Prior Work: Implementation of Research Electronic Data Capture at Wits FHS (2012-2013)

In 2012, as part of a process to strengthen research support, training, and outputs [8,24], the Wits FHS implemented REDCap (Research Electronic Data Capture; Vanderbilt University), a web-based EDC tool created by informaticists at the Vanderbilt University Medical Center (VUMC) in Nashville, Tennessee [8,25].

REDCap allows users to build electronic data collection instruments for a wide range of data types and environments. It is specifically geared toward research studies and operational data and toward enabling the capture and management of data in a manner that is compliant with 21 Code of Federal Regulations Part 11, the Federal Information Security Management Act, the Health Insurance Portability and Accountability Act, and General Data Protection Regulation [14,25]. Each project will have its own procedures for validation and quality control, and REDCap has many features available to end users to support this, such as granular user rights, a detailed audit log, and data quality control tools

One of the strategic goals of the Wits FHS was to unify and systematize health care and research data collection within the institution [8]. REDCap was an attractive option because of its freeware licensing model for noncommercial use and large international support community [14,25]. The decision to implement REDCap was also supported by an existing diaspora relationship between the Wits FHS and the VUMC [26].

The strategies used to install REDCap and overcome the initial implementation barriers within the Wits FHS have been presented previously [8]. The crucial factors highlighted in the paper were support from the Wits FHS management and the allocation of a modest budget for hosting infrastructure, systems administration, and recruitment of personnel from existing staff for end-user support. Support staff were initially allocated part time on a sliding scale of need, which allowed dedicated end-user support while limiting costs. The hardware costs were limited to servers and security certificates, which were housed at existing university data centers. Four months after the implementation, the number of REDCap users at the Wits FHS was 81, and after 12 months it had increased to 140. The total costs to provide a functional REDCap platform for the first year was <US $9000 [8].

### Goal of This Study

EDC implementation projects often fail after the initial deployment because of resistance from, and lack of adoption by, end users, even when leadership, infrastructure, and human resources are mobilized successfully [1,2,5,9,13,18]. The Wits FHS used various strategies to engage and support end users to overcome these challenges, and the period from 2013 to 2021 was characterized by sustained—sometimes exponential—growth in the demand for REDCap accounts and support services. The aim of this paper was to discuss the methods that helped to overcome barriers to adoption because we believe that these strategies may be applied in other low- to middle-income and resource-constrained environments where EDC implementation and adoption are subject to similar challenges. We measured the growth in REDCap use by increases in user accounts, projects, and publication metrics at the Wits FHS. The key success factors identified and discussed in detail in this paper are as follows:

- Top-down organizational support for EDC
- A proactive response and support team that can train and support users
- Continual development of the support team through mentoring and participation in national and international activities
- Maintaining visibility through promotion campaigns, networking events, and academic symposia
- Collaborating with, and learning from, established international partners
- Secure and reliable hosting

## Methods

### Adoption-Support Strategies

#### Hosting and Systems Administration

To gain the trust of users, the reliability of the Wits REDCap system was paramount. The system was deployed on 2 virtual machines—one for the MySQL database and a second one for the REDCap application—on a reliable Intel server with 64 GB of RAM and considerable disk space. The Ubuntu long-term support operating system version current at the time was used for both virtual host and physical machines. New hardware was introduced every 3 to 4 years; the cost implications when amortized over the life span of the machine were small. Older, retired hardware was recycled for less-critical work. Multiple levels of backup were used. Daily backups of the database and uploaded files were kept on a separate machine. Three times a week copies of the virtual machine images were created and stored on a server on a different campus. No major hardware failures occurred during this period, but tests were performed to emulate recovery using the backed-up data to ensure that this would be possible in the event that the primary hardware failed. Nagios software (Nagios Enterprises, LLC) [27] was used to monitor system health and stability. The electricity supply in South Africa was periodically unreliable; however, the server was placed in the university’s data center, with multiple backup power redundancies and physical security infrastructure. The REDCap application itself proved to be highly reliable, with regular bug fixes, security, and functionality updates released by the VUMC developers. The human resource allocation dedicated to system administration from 2013 to 2019 was equal to approximately 0.05 full-time equivalents (FTEs).

In 2019, the load created by concurrent users and processes made it necessary to move from magnetic to solid state drives. From 2020 onward, infrastructure and systems administration demands outgrew the existing resources. The Wits FHS REDCap system was moved from the Wits data center to a leading South African cloud hosting provider (Teraco) [28]. Additional systems administration capacity from the Wits Health Consortium [29], a wholly owned subsidiary of Wits, was brought in to manage the cloud hosting environment, with continued guidance and leadership from the original systems administrator. The Wits Health Consortium infrastructure team uses Arcserve [30] for daily snapshots of the virtual environment, with a separate backup every 1 to 3 days on a removable storage device for offsite storage. At the time of writing, the time spent on systems administration totaled approximately 0.1 FTE.

#### End-User Support

The second crucial component of the Wits FHS implementation strategy was a dedicated *go-to* individual to support end users, known as the REDCap administrator [8]. One of the most effective types of individuals to place in this role is referred to in the literature as a “technology bridger” [2,9,31]. A bridger is an early adopter who has a deep understanding of the technology being implemented as well as the soft skills to teach and support others at their organization.

The implementation of technology is a form of change management, and by approaching end-user support with an open-door policy and a culture of *psychological safety* (“a belief that one will not be punished or humiliated for speaking up with ideas, questions, concerns, or mistakes” [32]), the anxiety concerning, and resistance to, change exhibited by end users is reduced [personal communication by Wits REDCap administrator, July 2021].

By encouraging one-on-one consultations with the REDCap administrator in a relaxed and informal setting, new users felt safe to discuss their concerns or expose where they might have a lack of understanding. All email support and one-on-one consultations were provided at no cost. Over time, the need for additional project design and management services for larger and more complex projects became clear. To protect the REDCap administrator from users wanting to make use of the design service, rather than engaging with the administrator, to learn how to use the system on their own, an hourly fee was implemented for design services.

As the number of end users grew, so did the support and administrative needs (Textbox 1). During the period 2013-2014, a part-time REDCap administrator (0.5 FTE) was adequate. This was increased to 1.0 FTE from 2014, and a second full-time administrator was added in 2016. Additional part-time REDCap administrators were added in 2021 in response to a large increase in system use, currently totaling approximately 2.2 FTEs on average.

**Number of REDCap (Research Electronic Data Capture) application administrator full-time equivalents (FTEs) per year.**

**REDCap application administrator FTEs**

- 2013: 0.5
- 2014: 1.0
- 2015: 1.0
- 2016: 2.0
- 2017: 2.0
- 2018: 2.0
- 2019: 2.0
- 2020: 2.0
- 2021: 2.2

#### Hands-on REDCap Training

The REDCap application has a number of built-in tutorial videos and extensive *Help* and *Frequently Asked Questions* documentation. However, our experience showed that in addition to one-on-one consultations, the majority of late-adopting end users benefited from in-person formal participative instruction in the use of REDCap design tools. A series of sessions were offered in 2013, the content and format of which informed the creation of formal structured REDCap training workshops in 2014 and thereafter. Although the workshops were well attended, there was a significant proportion of attendees who had made reservations but did not attend. This prompted the introduction of a registration fee, which improved compliance and provided funds to contribute to the sustainability of the REDCap support team.

Initially, a basic introduction-to-REDCap workshop was offered, but as users became more skilled, a more advanced workshop was added in 2015. Good design practices and standardization of metadata were encouraged, and the workshops also established an interpersonal relationship between the end users and the REDCap administration team. A few groups, both within and external to the Wits FHS, requested on-demand REDCap training programs similar in content to that of the workshops. The number of REDCap training workshops and attendees are summarized in Table 1 and Table 2, respectively.

Each year, approximately 5 introductory and 4 advanced workshops were delivered, with an average of 19 and 8 attendees at each type of workshop, respectively. Step-by-step workshop manuals were also compiled iteratively over time and provided to attendees from 2017 onward. A total of 977 individuals attended all workshops between 2014 and 2020: 721 (73.8%) were Wits FHS–affiliated, whereas 256 (26.2%) were from external organizations. Fewer workshops were offered in 2018-2019 because of staffing constraints. From March 2020 onward, workshops were shifted to an internet-based platform (Zoom; Zoom Video Communications, Inc) because of COVID-19 restrictions on in-person gatherings. The availability of web-based teaching resulted in an increase in on-demand workshops—5 were held in 2020 compared with between 1 and 3 in previous years. We attribute this increase in part to the wider access inherent in the web-based format and partly because of the increased number of new users in 2020.

**Table 1:** The number of REDCap (Research Electronic Data Capture) workshops from 2014 to 2020, as recorded by web-based booking forms and attendance registers.

Workshops	2014	2015	2016	2017	2018	2019	2020
Introductory	2	6	6	7	5	5	5
Advanced^a^	0	2	3	5	3	4	3
On-demand	0	2	1	1	3	0	5
Total	2	10	10	13	11	9	13

^a^Before 2019, these workshops were referred to as *intermediate* hands-on *REDCap workshops*. A significant proportion of novice users attempted the intermediate sessions and found the content and pace of the intermediate session to be beyond their capacity. We therefore changed the name to *advanced* hands-on *REDCap workshops* and set up entry requirements in 2019 to emphasize that attendees had to have mastered the basics on their own or attended an introductory session before attempting the advanced one.

**Table 2:** The number of REDCap (Research Electronic Data Capture) attendees from 2014 to 2020, as recorded by web-based booking forms and attendance registers.

Attendees	2014	2015	2016	2017	2018	2019	2020
Wits FHS^a^	21	108	167	110	113	94	108
External	9	38	45	80	25	16	43
Total	30	146	212	190	138	110	151

^a^Wits FHS: University of the Witwatersrand Faculty of Health Sciences.

#### REDCap Consortium Participation

##### Overview

The *REDCap Consortium* is a community of REDCap administrative and technical support staff from the academic, nonprofit, and government institutions that have adopted REDCap [14]. The REDCap Consortium represents a *professional home* for many of the local REDCap research informatics leaders, and it is a forum for enabling teams to share ideas, problems, and solutions related to the innovative use of REDCap [14]. Feedback and communication with the local administrators participating in the REDCap Consortium is a vital component of how REDCap program leaders understand unmet needs, socialize concepts for new features, and eventually prioritize new development. The voluntary participation in the REDCap Consortium is a very valuable investment for organizations [14,33]. Membership of the consortium gives partner organizations’ REDCap administrators access to various networking and information-sharing platforms, of which there are three main types: (1) consortium calls, (2) conferences and symposia, and (3) the REDCap Community forum. Between late 2013 and April 2020, the number of REDCap Consortium members from Africa and South Africa grew from 26 and 8, respectively, to 261 and 175, respectively [8,34].

##### REDCap Consortium Calls

The REDCap software development team at the VUMC hosts a weekly technical call, a forum to share news and updates and generally bring the REDCap developer and administrator community up to date with the latest REDCap features. Since 2016, the VUMC has also hosted 2 Eastern Hemisphere Partner consortium calls at times that made them accessible to the Africa and Europe as well as Australia, New Zealand, and Japan regions. Various subcommittees have also been formed within the REDCap Consortium, either based on common interest (software validation or development of training materials) or on shared geographical location and language (Hispanophone or Francophone committees). Some of these subcommittees also host regular calls relating to their specific domains. A locally hosted call for the African region was added in 2018 through a collaboration between the REDCap administrator of the Pan-African Bioinformatics Network for Human Heredity and Health in Africa, based at the University of Cape Town, and the Wits REDCap team. Participation in these calls supports and develops REDCap administrators by keeping them up to date with the latest developments, enabling networking and exchange of ideas, as well as giving them a platform to connect directly with the REDCap software development team at the VUMC.

##### Conferences and Symposia

The annual REDCap conference (REDCapCon) is a forum for REDCap administrators from different countries, institutions, and environments to meet, share experiences, and create a support network. The opportunity to interact with international members forms the basis of a collective resource for information dissemination and problem solving within the global REDCap Community. Participation in the annual REDCapCon has proved very valuable in terms of the opportunity to attend and present our work. A representative from Wits FHS has attended the annual REDCapCon since 2015. However, many sub-Saharan Africa–based REDCap administrators do not have a budget to travel to North America for the annual REDCapCon. It became apparent that an African REDCapCon would add value to the African consortium partners. Wits hosted the first REDCap Africa Day in Johannesburg in 2016 as an adjunct to the FHS research day, followed by 3 more REDCap Africa symposia in 2017, 2019, and 2020 (Table 3). Each symposium has been attended by one or more members of the VUMC REDCap team. The REDCap Africa event encourages attendance by regional and international REDCap administrators as well as end users, as opposed to the REDCapCon, where only administrators attend. The REDCap Africa symposia agendas were a mixture of technical presentations and use cases, with plenary sessions presented by local academics as well as VUMC visitors. Delegates included local and regional faculty members, research institute employees, and students.

The novelty of the first REDCap Africa symposium drew large numbers of attendees (Table 3), including a number of casual attendees from the Wits FHS who were not REDCap users but nonetheless wanted to learn more about REDCap, the EDC strategy of the Wits FHS, and the relationship with the VUMC. In subsequent years, the number of casual attendees decreased and was made up of REDCap administrators or highly engaged power users. The cost of intra-Africa travel is high, and the number of attendees from the African region who were able to attend in person remained low. In 2020, because of COVID-19 restrictions, REDCap Africa Day was hosted using Zoom, and attendance was significantly higher with more international delegates than at any previous event. In October 2021, again because of the ongoing COVID-19 pandemic, we organized another Zoom-based installment of REDCap Africa Day (we have institutional review board clearance to report data up to September 30, 2021; hence, we cannot report the actual number of attendees), but future REDCap Africa symposia will explore combined in-person and livestreaming as well as travel bursaries to encourage regional participation.

**Table 3:** The number of delegates to the REDCap (Research Electronic Data Capture) Africa symposia per year.

	2016	2017	2019	2020
Wits FHS^a^ affiliated delegates	69	40	27	42
Local (SA^b^) delegates	30	28	19	37
International delegates	6	2	4	31
Total number of delegates	105	70	50	110

^a^Wits FHS: University of the Witwatersrand Faculty of Health Sciences.

^b^SA: South Africa.

##### REDCap Community Forum

The REDCap Community is a web-based platform where the administrative and IT support staff of a consortium partner institution can access software downloads, extensive technical documentation, a question-and-answer forum, consortium announcements, committee activities, events, and more [35]. The REDCap Community website provides a forum for interaction on, and dialogue about, REDCap-related topics with REDCap administrators around the globe, and it is an essential resource for the development of an institution’s capacity to host and support REDCap. The Wits FHS REDCap administrators have received technical advice or otherwise benefited from discussions on the REDCap Community forum, while also enjoying the networking experience and the sense of community gained by interacting with peers from other institutions.

#### VUMC Relationship and Support

The development of clinical research informatics capacity and health care IT skills are particularly important in resource-constrained settings such as sub-Saharan Africa, and the Wits-VUMC partnership has contributed to the capacitation process at the Wits FHS. Initially, the relationship between Wits and the VUMC grew from an alumni diaspora program initiated in 2010 [36]; it later expanded through bilateral visits by academic staff [26], custom REDCap development projects, joint grant applications, and mentorship. New REDCap Consortium members in resource-constrained environments may benefit from leveraging existing diaspora linkages or from initiating new mentorship and capacity-development collaborations with institutions, such as the VUMC, that have mature research informatics divisions.

### Measurement of System Use and Growth

#### Overview

REDCap system use is reported in 2 main ways: the number of users and the number of projects. The number of users and projects on any REDCap system is available on the application’s administration web page called the *Control Center*. The Wits FHS REDCap administrator has a monthly record of several use metrics since system installation in August 2012. Other metrics were determined retrospectively by running MySQL queries on the REDCap database logs with the help of a REDCap external module named *MySQL Simple Admin* [37]. These queries were used to identify and enumerate project- or user account–creation events. The descriptive system-use metrics that we report on are as follows:

1. Total number of user accounts
2. Number of user accounts that were active each year
3. Annual increase or decline in number of active user accounts
4. Total number of practice and nonpractice projects
5. Project-purpose attribute of each nonpractice project

#### User Accounts

A REDCap user account allows an individual user to access their own private repository of projects and data. The REDCap Control Center displays various metrics regarding accounts; for example, the total number of user accounts, the number of accounts that were active in a given period, and the number of accounts that were suspended because of inactivity. Activity is defined as logging in and performing an action on REDCap. At the Wits FHS, the period of inactivity that leads to suspension is 180 days (this may differ from institution to institution based on policy). We used a combination of Control Center statistics and MySQL Simple Admin queries to report the total number of user accounts on the Wits FHS REDCap system per year as well as the number of accounts that were active in a given year. The measurement is taken on the first day of September each year.

In addition, the growth and decline in the number of active user accounts were determined by taking the number of active users for a given year and subtracting the number of active users from the previous year.

#### Projects

A *project* in REDCap refers to a set of connected electronic data collection screens and records that are related to a specific purpose.

When creating a project, end users are required to allocate one of five possible project purposes, namely *practice*, *research*, *quality improvement*, *operational support*, or *other*. The Control Center report usually excludes practice projects from the *total projects* metric, but we performed a query using MySQL Simple Admin to retrieve the number of practice projects as well, and we report these together with the total nonpractice projects. We believe that practice projects are an important metric of how comfortable users are to experiment and explore the system, something which is actively encouraged by our REDCap administrators during one-on-one or group training sessions.

Furthermore, we performed an annual breakdown of the nonpractice projects by purpose, and report the percentage of projects in the categories of *research*, *quality improvement*, *operational support* and *other*.

### Measurement of System-Related Research Outputs

One way to measure the impact of REDCap on the Wits FHS is by reviewing research outputs such as journal articles and postgraduate theses. We performed a bibliometric survey to determine the number of research outputs from Wits FHS–affiliated authors that relied on the use of Wits FHS REDCap for EDC or data management and report the number of outputs per year as well as the subject domains across all the outputs for the period from 2013 to 2020. REDCap has a built-in publication-matching tool that will review PubMed databases for authors and affiliations that match those of REDCap users or project principal investigators. We evaluated the list of *potential matches* generated within REDCap and only included articles and monographs in our results if they mentioned using Wits FHS REDCap as the data collection instrument in the methods or acknowledgments sections. In cases where no system was mentioned by name, we contacted the authors through email to request clarification and only included the output if authors confirmed in writing that Wits FHS REDCap was used.

### Ethics Approval

Permission to perform the research was obtained from the Wits Department of Surgery postgraduate protocol committee, the Wits Human Research Ethics Committee (M210551), and the Wits University Registrar.

## Results

### User Accounts

**Figure 1:**
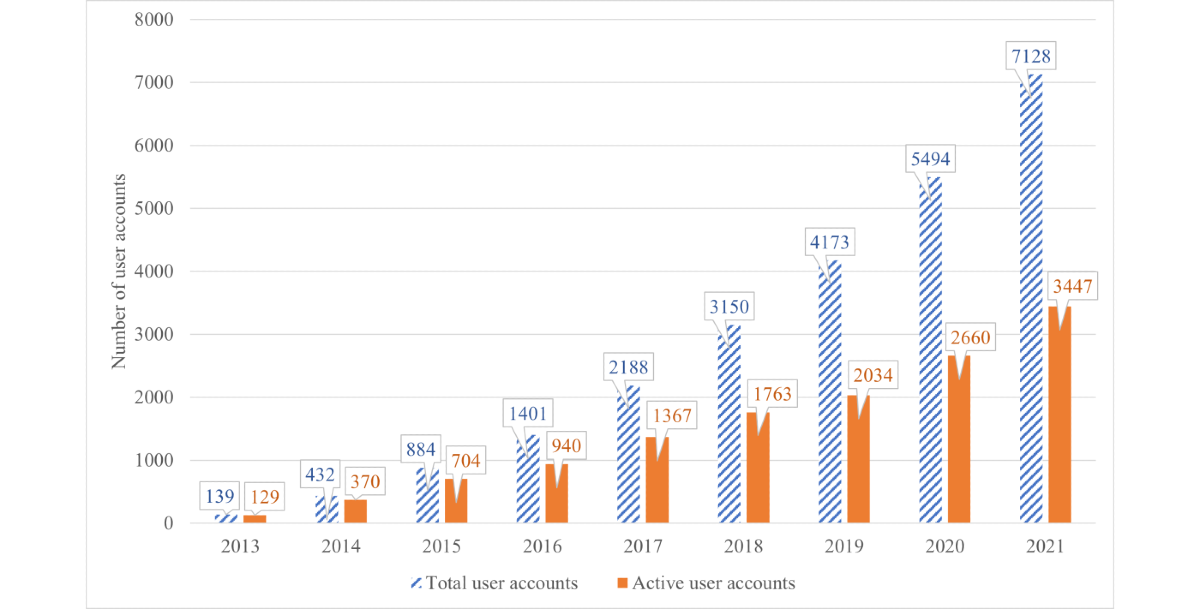
User account statistics for the University of the Witwatersrand Faculty of Health Sciences REDCap (Research Electronic Data Capture) system from September 2013 to September 2021. The total number of accounts as well as the active cohort is shown for each year.

There was a sustained—and at times exponential—increase in the number of user accounts from 139 total and 129 active accounts in 2013 to 7128 total and 3447 active accounts in 2021 (Figure 1).

**Figure 2:**
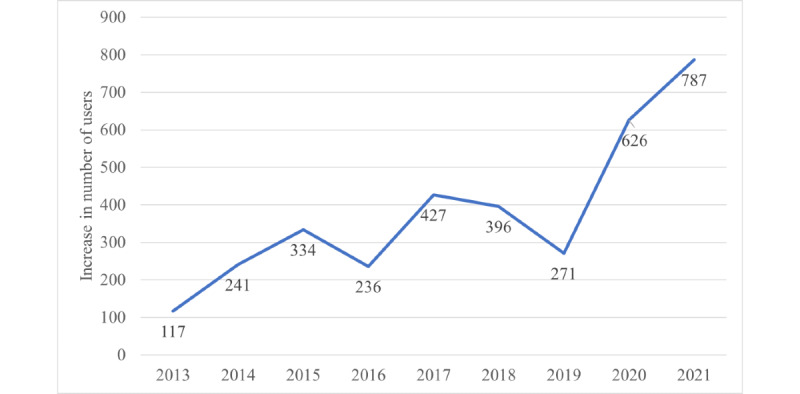
The increase in the number of active REDCap (Research Electronic Data Capture) users at the University of the Witwatersrand Faculty of Health Sciences from 2013 to 2021. The values were obtained by taking the number of active users in a given year and subtracting the number of active users recorded for the previous year.

The number of active users has increased 25-fold in 8 years; however, the magnitude of the growth was variable (Figure 2). Except for the 2016 and 2018-2019 periods, annual growth exceeded 25%.

### Projects

**Figure 3:**
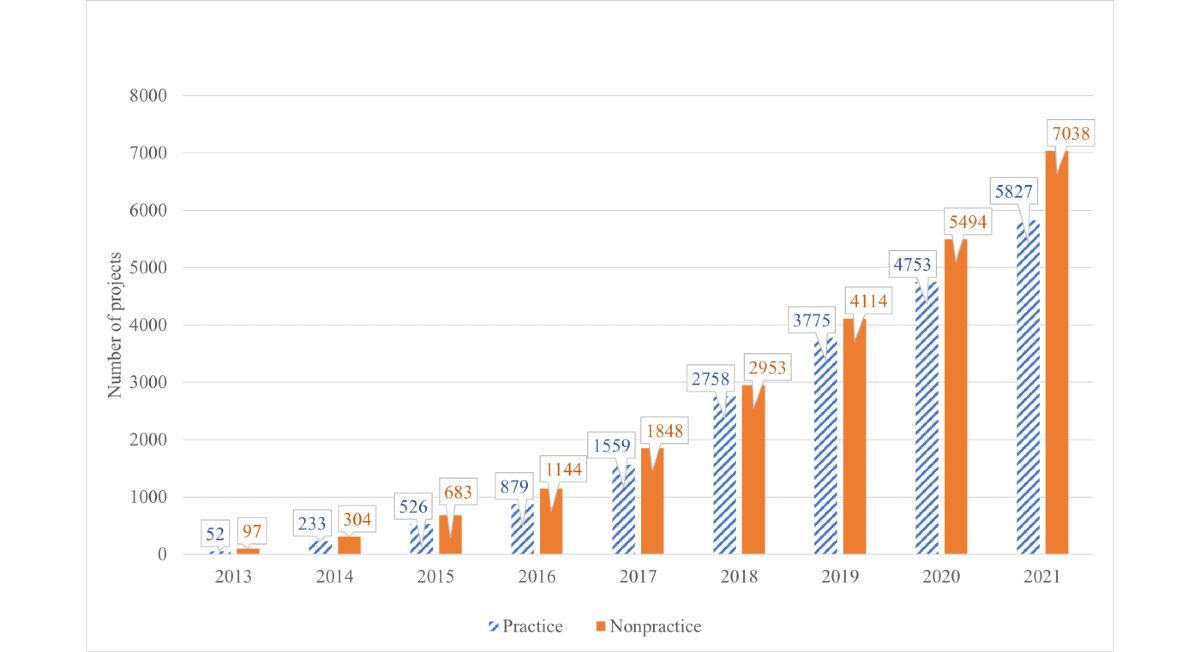
REDCap (Research Electronic Data Capture) projects on the University of the Witwatersrand Faculty of Health Sciences system per annum from September 2013 to September 2021.

The total number of projects on the Wits REDCap platform increased significantly from 149 in September 2013 to 12,865 in September 2021 (Figure 3). The number of nonpractice projects increased from 97 in September 2013 to 7038 in September 2021 and accounted for 54.71% (7038/12,865) of the total number of projects in 2021.

Of the 7038 nonpractice projects on the Wits REDCap system in September 2021, the majority (n=3952, 56.15%) were for research purposes (Table 4), and the remaining (n=3086, 43.85%) were dedicated to nonresearch purposes (Table 4).

The majority of the Wits REDCap nonresearch projects in 2021 were allocated as *operational support* (1850/3086, 59.95%), whereas *quality improvement* and *other* represented a minority (705/3086, 22.84%, and 531/3086, 17.21%, respectively).

From Table 4, it can be seen that the *operational support* category has grown to represent a larger share of the total projects every year (from 10/97, 10%, in 2013 to 1850/7038, 26.29%, in 2021), whereas the research, quality improvement, and other categories have all declined as a percentage of the total.

**Table 4:** The number of nonpractice projects on the University of the Witwatersrand Faculty of Health Sciences REDCap (Research Electronic Data Capture) system from September 2013 to September 2021, categorized by purpose.

	Research, n (%)	Operational support, n (%)	Quality improvement, n (%)	Other, n (%)
2013 (n=97)	62 (63.92)	10 (10.31)	16 (16.5)	9 (9.28)
2014 (n=304)	209 (68.75)	36 (11.84)	38 (12.5)	21 (6.91)
2015 (n=683)	452 (66.18)	79 (11.57)	88 (12.88)	64 (9.37)
2016 (n=1144)	757 (66.17)	170 (14.86)	116 (10.14)	101 (8.83)
2017 (n=1848)	1223 (66.18)	324 (17.53)	169 (9.15)	132 (7.14)
2018 (n=2953)	1842 (62.38)	593 (20.08)	265 (8.97)	253 (8.57)
2019 (n=4114)	2523 (61.33)	887 (21.56)	392 (9.53)	312 (7.58)
2020 (n=5494)	3145 (57.24)	1376 (25.05)	558 (10.16)	415 (7.55)
2021 (n=7038)	3952 (56.15)	1850 (26.29)	705 (10.02)	531 (7.54)

### Wits REDCap Publication Metrics

**Figure 4:**
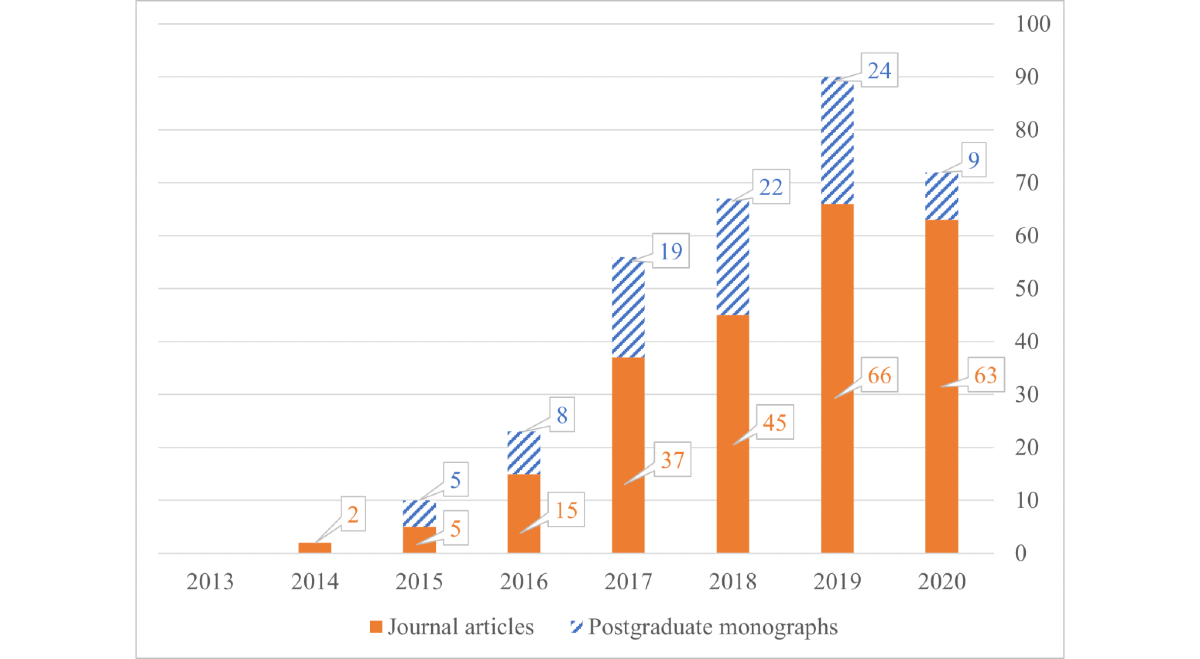
The number of research outputs linked to use of the University of the Witwatersrand Faculty of Health Sciences REDCap (Research Electronic Data Capture) system, grouped by publication year. Postgraduate monographs include PhD and master’s research works. Certain data included herein are derived from Clarivate Web of Science (copyright Clarivate 2021; all rights reserved).

In total, 233 journal articles and 87 postgraduate research monographs acknowledging the use of the Wits FHS REDCap system were published between 2013 and 2020. As shown in Figure 4, the number of articles increased over time as more users adopted the system and as projects reached maturity and results were disseminated. The year 2020 saw a sharp decrease in postgraduate monographs that cite the Wits FHS REDCap system. This may be due to a delay before a thesis or dissertation becomes available on the institutional repository. Additional delays in postgraduate submissions could be a result of strict COVID-19 shutdowns in South Africa.

**Figure 5:**
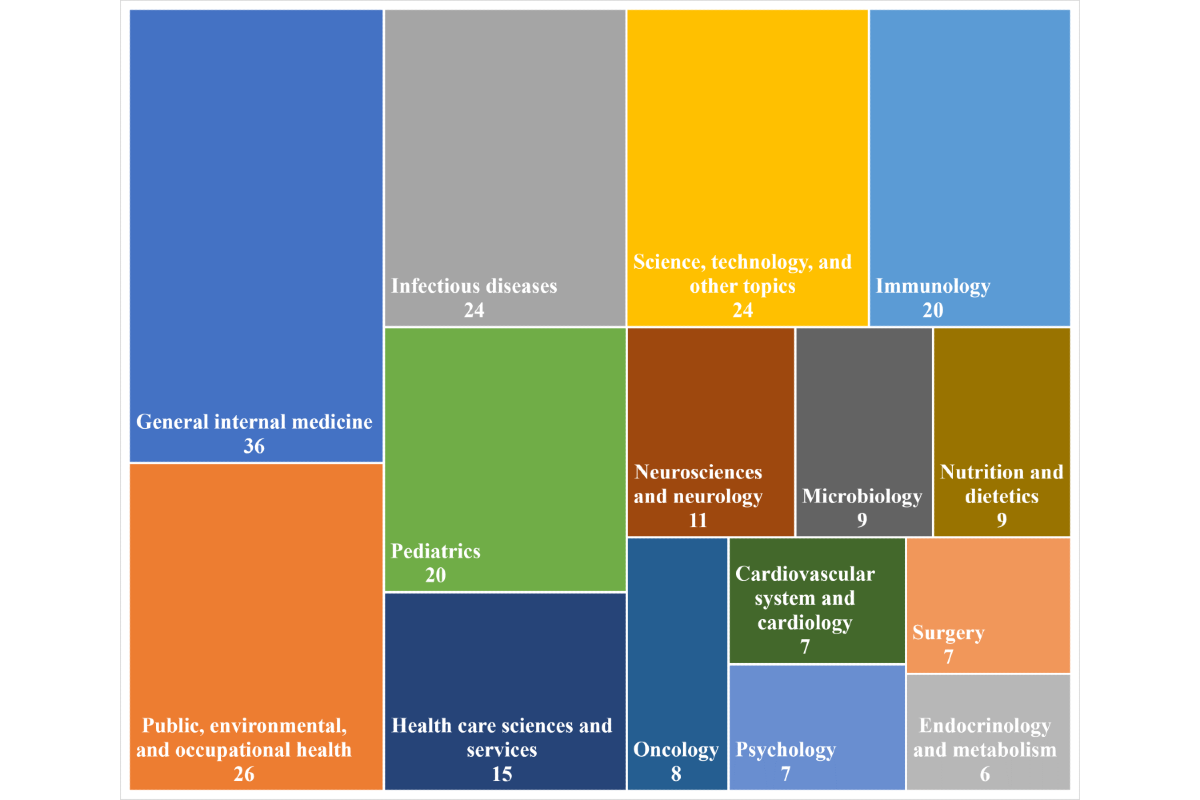
Treemap visualization showing the Witwatersrand Faculty of Health Sciences publications between 2013 and 2020 that were supported by REDCap (Research Electronic Data Capture), categorized by research area. This graph represents the top 15 out of 50 research areas by frequency of occurrence. Certain data included herein are derived from Clarivate Web of Science (copyright Clarivate 2021; all rights reserved).

A visualization of the research areas of the journal articles also illustrates the diversity of scientific disciplines on which REDCap at the Wits FHS had an impact (Figure 5).

## Discussion

### Principal Findings

During the period 2012-2013, the Wits FHS implemented the REDCap EDC system to support both research and clinical service delivery data management needs [8]. REDCap provides researchers with the means to design and develop EDC tools that conform with international best practices for the safety, security, and privacy of clinical data. REDCap is licensed by Vanderbilt University at no charge to government, academic, and nonprofit organizations for use in noncommercial academic and research contexts [14,25]. By removing financial burden and providing tools and support channels that empower local research informatics leaders to serve the local research enterprise, the REDCap platform fills a critical gap in most research organizations [14,25].

During the period from 2013 to 2021, use of the REDCap EDC system increased steadily at the Wits FHS, as evidenced by the growth of users, projects, and publications.

The number of active Wits FHS REDCap users increased from 129 in 2013 to 3447 in 2021, which is a 25-fold increase. Each year saw more active users than the preceding year, and with the exception of 2016, 2018, and 2019, the annual increase in the number of users was larger every year. Although our records do not contain an explanation for the slower growth in 2016, the 2018-2019 period saw fewer hands-on workshops being offered because of the senior REDCap administrator being on extended leave. In 2020 and 2021, the annual increase was 1.5-fold and 2-fold higher, respectively, than in any previous year. Two factors may have contributed to the 2020-2021 surge in active users: first, the COVID-19 pandemic drove the adoption of web-based instruments that could be accessed by teams working remotely, and second, the Protection of Personal Information Act (POPIA) was introduced in South Africa in 2020 and enforced after July 2021 [38]. As part of POPIA requirements, organizations that process personal information were required to use secure, auditable applications, and REDCap was one of only a few products offered by our institution that were compatible with POPIA requirements.

The total Wits FHS REDCap projects numbered 149 in 2013 and 12,865 in 2021. Of the 12,865 projects in 2021, a total of 5827 (45.29%) projects were created for *practice* purposes, and 7038 (54.71%) projects were for nonpractice purposes. The ratio of practice to nonpractice projects has remained remarkably stable at approximately 45% over time. Practice projects are created during the formal hands-on training workshops offered by the Wits FHS, and the use of practice projects to test design ideas and prototype data collection instruments are encouraged by the Wits FHS REDCap administrators during one-on-one consultations. The REDCap application developers release updated features on an almost monthly basis, meaning that even experienced users might resort to creating practice projects from time to time to test out new functionality. A deeper analysis of user behavior may be needed to determine conclusively the reason for the observed stability of the ratio of practice to nonpractice projects.

Of the 7038 nonpractice projects on the Wits REDCap system in September 2021, the majority were for research purposes (n=3952, 56.15%; Table 4), whereas the remaining (n=3086, 43.85%) projects were dedicated to nonresearch purposes (Table 4). This reflects the diversity of the uses of REDCap in the Wits environment: as a clinical health record and a staff rostering and management tool, as well as in a multitude of *spreadsheet*-type administrative processes, in addition to research. Further innovative off-label uses of REDCap appear in the literature [39-42].

During the period from 2013 to 2020, a total of 233 papers and 87 postgraduate monographs that acknowledge the use of Wits FHS REDCap were published. The increase in research outputs occurred during a time when the Wits FHS research and postgraduate support office used several strategies to increase research and publication rates [24], and REDCap was one of the contributing factors to the observed rise in publication metrics of Wits FHS staff and students.

### EDC-Support Strategies in a Resource-Constrained Environment

The growth in the use of REDCap at Wits FHS was driven in large part by the trust generated by offering a reliable, secure service and a strong end-user training and support model (Textbox 2). A critical success factor is that hosting and server infrastructure was supported by a highly experienced systems administrator who ensured that appropriate security measures and disaster recovery plans were in place. Additional storage and computing capacity was added as needed. It was important to respond to performance problems when incremental upgrades were no longer sufficient. End users were supported by a dedicated REDCap administrator available through an email helpdesk, one-on-one consultations, formal training workshops, and annual symposia. The support team size was expanded over time to meet the increase in demand (REDCap administrator or administrators: from 0.5 FTEs in 2013 to 2.2 FTEs in 2021 and systems administrator or administrators: from 0.05 FTEs in 2013 to 0.1 FTEs in 2021). The capacity of the support staff was improved through mentorship, professional development, and participation in regional and international REDCap Consortium activities.

**Summary of support strategies for electronic data capture (EDC) adoption at the University of the Witwatersrand Faculty of Health Sciences.**

**Support strategy and observations and effects**

- Top-down organizational support
  - Official endorsement from management ensures that an EDC system receives adequate resources for infrastructure and staff and signals institutional commitment to potential end users.
- Secure and reliable application, hosting infrastructure, and systems administration
  - Prioritizing reliability and security of the EDC system builds trust among users. In resource-constrained settings, power, network, and information infrastructure is often unreliable, and users fear losing their data.
- An enabling and accessible REDCap (Research Electronic Data Capture) support team
  - The availability of a person or team acting as technology bridgers [31] reduces anxiety and resistance associated with technology adoption.
- Regular hands-on training workshops
  - Structured practical training opportunities capacitate new users with an EDC system and serve as a mechanism to disseminate knowledge and best practices.
- Annual conferences or symposia for end users
  - Regular academic events that promote the correct use of features and demonstrate the benefits of an EDC system attract new users while keeping existing users informed and engaged.
- Participation in international REDCap Consortium activities
  - REDCap administrators’ knowledge and abilities are developed through interaction with peers from other international institutions.
- Mentorship- and capacity-development relationships with established organizations
  - Institutions that do not have established clinical research informatics departments or capacity benefit greatly from mentorship and collaboration with experienced partner institutions.

### Gap Analysis

Although adoption has been a success, there were gaps in our processes, which are important to recognize, especially for those starting the process. Although top-down organizational support has been strong, funding has always been difficult in a resource-constrained environment. A user-pays model can be attractive, but implementation is difficult and may encourage users to stick to paper and spreadsheets. Funding from the center may be politically fraught, especially in the early phases before the system has proved itself. We have gained stability over the last 10 years but are still working on improving the financial model. A related issue is the size of the support team—dedicated staff are required, and in resource-constrained environments this may be hard to find or pay for. In our case, we were fortunate to have dedicated staff members from the start, but they were always working under pressure, which limited the extent of training and ability to support strategic projects. This latter issue was mitigated to some extent by the strong mentoring role played by the VUMC. Investment in the support team through attendance at international events has been very important, but resource constraints have limited how many individuals can attend and how frequently.

### Limitations of This Study

The principal investigator on this study (IM) is also the lead REDCap administrator for the Wits FHS. This makes them intimately familiar with the support processes and end-user interactions at the institution but can lead to a lack of objectivity. For this reason, coauthors experienced with REDCap and clinical research informatics from outside of our institution were included to provide a more balanced and fair report.

### Conclusions

The implementation of technology requires strategies to support, and manage resistance from, end users. One of the main reasons for this resistance is inertia: end users naturally resist change to familiar and deeply ingrained processes [1,5,7,15-17]. In our experience, and supported by findings from others [1,4,7,16,17], to overcome inertia, one needs to demonstrate the reliability of the system and benefits of adoption to prospective users, while at the same time easing the transition process by providing adequate end-user support. The capacity to implement and support REDCap at the Wits FHS was initiated through organizational and financial backing of the FHS management. This capacity was subsequently developed further through participation in REDCap Consortium activities such as REDCapCon and the REDCap Community forum and through a strong bidirectional relationship with the VUMC, the institution that created REDCap.

## Abbreviations

EDC: electronic data capture
FTE: full-time equivalent
POPIA: Protection of Personal Information Act
REDCap: Research Electronic Data Capture
REDCapCon: Research Electronic Data Capture conference
VUMC: Vanderbilt University Medical Center
Wits FHS: University of the Witwatersrand Faculty of Health Sciences
